# Normal Histology

**Published:** 1911-01

**Authors:** 


**Normal Histology.** By **George A. Piersol,** M.D., Sc.D., Professor of Anatomy in the University of Pennsylvania. **Eighth edition.** Octavo, pp. 426. Illustrated. Philadelphia and London: J. B. Lippincott Co. (Cloth, $3.50.)

The author has taken advantage of the opportunity given him by a call for the eighth edition of his book to completely rewrite, rearrange, and add much new material to this issue

From the fact that histology has its place at the beginning of the medical curriculum, and the student is often required to study the microscopic details of organs as he becomes acquainted with their gross anatomy, a careful and frequent revision of the subject of histology becomes necessary on account of increased study and experiment along this line. In this volume only such methods are recommended upon which experience has proved that dependence can be placed, and the directions given are for the most part capable of ready modification to accord with the ideas of different teachers. There are 438 illustrations, many of which are in colors. All are helpful and aids in the study of a subject which to most students is dry and tedious. J. A. R.

